# Assembly of root-associated N_2_O-reducing communities of annual crops is governed by selection for *nosZ* clade I over clade II

**DOI:** 10.1093/femsec/fiac092

**Published:** 2022-08-04

**Authors:** Daniel R H Graf, Christopher M Jones, Ming Zhao, Sara Hallin

**Affiliations:** Department of Forest Mycology and Plant Pathology, Swedish University of Agricultural Sciences, Box 7026, 75007 Uppsala, Sweden; Department of Forest Mycology and Plant Pathology, Swedish University of Agricultural Sciences, Box 7026, 75007 Uppsala, Sweden; Department of Plant Biology, Swedish University of Agricultural Science, Box 7080, 75007 Uppsala, Sweden; Department of Forest Mycology and Plant Pathology, Swedish University of Agricultural Sciences, Box 7026, 75007 Uppsala, Sweden

**Keywords:** denitrification, nitrous oxide, rhizosphere, soil

## Abstract

The rhizosphere is a hotspot for denitrification. The nitrous oxide (N_2_O) reductase among denitrifiers and nondenitrifying N_2_O reducers is the only known N_2_O sink in the biosphere. We hypothesized that the composition of root-associated N_2_O-reducing communities when establishing on annual crops depend on soil type and plant species, but that assembly processes are independent of these factors and differ between *nosZ* clades I and II. Using a pot experiment with barley and sunflower and two soils, we analyzed the abundance, composition, and diversity of soil and root-associated N_2_O reducing communities by qPCR and amplicon sequencing of *nosZ*. Clade I was more abundant on roots compared to soil, while clade II showed the opposite. In barley, this pattern coincided with N_2_O availability, determined as potential N_2_O production rates, but for sunflower no N_2_O production was detected in the root compartment. Root and soil *nosZ* communities differed in composition and phylogeny-based community analyses indicated that assembly of root-associated N_2_O reducers was driven by the interaction between plant and soil type, with inferred competition being more influential than habitat selection. Selection between clades I and II in the root/soil interface is suggested, which may have functional consequences since most clade I microorganisms can produce N_2_O.

## Teaser

Plant roots recruit specific communities of nitrous oxide reducing microbes from the surrounding soil, with contrasting ecological processes determining the structure of these communities in root vs. soil.

## Introduction

Denitrification is a major route for loss of nitrogen from ecosystems. In this facultative anaerobic respiratory process, nitrogen oxides are reduced to dinitrogen (N_2_) gas by a diverse range of mainly bacterial taxa (Philippot et al. 2007, Graf et al. 2014). However, denitrification is a modular process in that organisms can have different subsets of enzymes (Graf et al. 2014), which has an effect on the production and reduction of the greenhouse gas nitrous oxide (N_2_O; Philippot et al. 2011, Domeignoz-Horta et al. 2016). Incomplete denitrification, either due to regulation or lack of the N_2_O reductase, is a major source of N_2_O (Conrad 1996, Philippot et al. 2011) and the largest emissions are from agricultural soils (Shcherbak et al. 2014). Denitrification is stimulated by plant-derived organic carbon and fluctuating levels of oxygen availability in the root compartment (Henry et al. 2008, Philippot et al. 2009), and a higher proportion of denitrifiers relative to other heterotrophic bacteria is generally detected in proximity to roots (Hamonts et al. 2013). Recent work suggests that microorganisms or consortia of microorganisms capable of complete denitrification with enzymatic reduction of N_2_O to N_2_ by the N_2_O reductase are selected for or stimulated around roots (Langarica-Fuentes et al. 2018, Graf et al. 2019, Ai et al. 2020).

The N_2_O reductase is encoded by the gene *nosZ*, which is phylogenetically separated into clades I and II (Sanford et al. 2012, Jones et al. 2013). Most organisms with *nosZ* clade II are nondenitrifying N_2_O reducers, whereas denitrifiers with N_2_O as an intermediate dominate clade I (Graf et al. 2014). The soil N_2_O sink capacity has been explained by the abundance and diversity of clade II in arable soils (Jones et al. 2014, Domeignoz-Horta et al. 2015, Yin et al. 2020) and nondenitrifying N_2_O reducing bacteria can lower the net N_2_O production from soil (Domeignoz-Horta et al. 2016). Both clades I and II are abundant in soils and rhizosphere (Hallin et al. 2018), but little is known if plants select for specific N_2_O-reducing communities and the underlying ecological processes governing the colonization of roots by N_2_O-reducers. Agricultural soils with annual crops and inversion tillage constitute a habitat where the root compartment develops every year and hence, the assembly of N_2_O-reducing communities around the root reoccurs with every new crop. A better understanding of the assembly processes of the root-associated N_2_O-reducing communities in agricultural systems is warranted since root surfaces and the rhizosphere are hot-spots for denitrification and denitrifying bacteria (Kuzyakov and Blagodatskaya 2015, Moreau et al. 2019).

We hypothesized that the composition of N_2_O-reducing communities around roots depends on soil type and plant species, as shown for bacteria in general (e.g. Berg and Smalla 2009, Bulgarelli et al. 2012), but that the assembly processes are general, irrespective of these factors and differ between clades I and II N_2_O reducing communities. As different environmental factors may influence community assembly processes to a lesser or greater degree depending on the scale at which local and regional communities are defined (Dini-Andreote et al. 2015), we established our scale of interest as the complete root compartment in contrast to the surrounding bulk soil. To test whether the assembly of N_2_O-reducing communities is governed by niche-based or neutral processes, we set up a pot experiment with two different soil types in which barley (*Hordeum vulgare*) and sunflower (*Helianthus annuus*), respectively were cultivated. Using a phylogenetic approach, we investigated the abundance and community structures of *nosZ* clade I and *nosZ* clade II in soil and in association with roots after 24 days of plant growth to infer the initial processes underlying N_2_O-reducing community assembly on the roots of annual crops during the vegetative state.

## Material and methods

### Experimental set-up and sampling of roots and soil

Soil was collected from two agricultural fields, Ekhaga and Kungshamn, located close to Uppsala, Sweden. Ekhaga (59 49 50.8 N, 17 48 16.9 E) is a clayey soil with a more neutral pH (31% clay, 50.5% silt, 9% sand, 4.3% total C, 0.33% organic N, 9.5% soil organic matter, and pH 6.6) whereas Kungshamn (59 46 48.8 N, 17 39 42.4 E) is a sandy loam, i.e. more acidic (16.4% clay, 29.1% silt, 53.3% sand, 1% total C, 0.07% organic N, 1.1% soil organic matter, and pH 5.6). Sampling was done outside the growing season when the soil was bare, and the soils were homogenized and sieved (2 mm) prior to establishing the pot experiment. The soils were selected after screening several soils. Our criteria were that intact root sampling should be possible (i.e. we avoided heavy clay soils) and that the sieved soil maintained a structure that prevented soil compaction and ensured proper root development. The final two soils were selected based on these technical criteria, as well as having contrasting edaphic properties.

A total of two food crops with different root traits were used in the pot experiment. *Hordeum vulgare* ‘Triple’ (barley) and *H. annuus* IREGI-type (sunflower), representing a mono- and dicotyledon, respectively, were selected based on differences in root morphology. The major difference is that barley has a fibrous root system with lateral roots and root hairs, whereas sunflowers have a tap root and secondary roots with root hairs. Prior to the experiment, we tested that they had similar rate of germination and growth to be able to synchronize harvest. The seeds were surface sterilized by washing twice with 5% bleach and 70% ethanol. For each soil, 15 pots (10 × 10 × 20 cm) were first filled with a 2-cm thick drainage layer of sterilized Lightweight Expanded Clay Aggregate (LECA), followed by either of the two soils. For each soil type, five replicate pots were sown with either barley, sunflower, or left unsown as a control containing only soil. After sowing, the surface was covered with a 2-cm deep layer of sterilized perlite. Pots were randomized on trays and placed in a growth chamber at the Phytotron facility at the Swedish University of Agricultural Sciences. The initial temperature was set at 20°C to facilitate germination. Immediately after germination, the pots were thinned to a density of five plants per pot for barley and three plants per pot for sunflower. The day and night temperatures were set to 20°C and 15°C, respectively, and day length was 18 h with optimal light conditions. Weeds germinating from seeds naturally present in the experimental soils were removed continuously. Soil moisture was monitored using a soil hydrometer (Sinometer, Shenzhen, China) and plants were watered when necessary to maintain 50%–70% of the maximum water retention capacity.

Soil and roots were destructively sampled after 24 days of growth when both species were still in vegetative stage. The selected time point for sampling was chosen to allow microbiome selection (Edwards et al. 2015, PNAS) and at the same time avoid potential negative plant feedbacks that may occur in pot experiments over time, e.g. due to nutrient depletion. Further, according to reported root exudate levels after 21–30 days of plant growth in both barley (Suku et al. 2014, Giles et al. 2017) and sunflower (Bowsher et al. 2015, Yang and Pan 2013), the amount of root exudate present should be substantial in our experiment. Plant development, with respect to number of leaves and height, was similar among replicates within and across pots according to visual inspection.

We took samples from two compartments at the soil–root interface; the soil still adhering to the roots after removal of plants from the bulk soil in the pots (defined as soil), and the root-associated zone consisting of washed root material. This compartment thus comprises both the rhizoplane and endosphere. The entire root system was sampled for all specimens (five for barley; three for sunflower) in each pot and then pooled at the pot level to minimize effects of any variation among individual plants. For the soil samples, soil was removed from the root system by vigorous shaking and gentle removal by hand. The soil was then mixed well and stored at −20°C until further analyses. The root-associated compartment was sampled by first washing roots in sterile phosphate buffered saline (137 mM NaCl, 2.7 mM KCl, 10 mM Na_2_HPO_4_, and 2 mM KH_2_PO_4_), then cutting roots into 1-cm pieces. A subsample was taken for potential N_2_O production rates and DNA extraction and stored at −20°C. Soils in the unplanted controls were removed from the pots, mixed thoroughly, then also stored at −20°C.

### Potential N_2_O production rates

Data on potential N_2_O production rates (Graf et al. 2016) were incorporated as an indication of availability of N_2_O. Briefly, rates were determined using 10 g of soil and 1.5 g of root samples placed in 147 and 32 ml flasks, respectively. The soil samples were incubated as a slurry with 20 ml distilled water and a final concentration of 3 mM KNO3, 1.5 mM succinate, 1 mM glucose, and 3 mM acetate at 25°C under anoxic conditions (nitrogen gas in the headspace) during 3.5 h. The roots were incubated with 6 ml water during 7 h, but otherwise under the same conditions. Gas samples from the headspace were taken every 30 min and N_2_O concentrations were determined using a gas chromatograph (Clarus-500, Elite-Q PLOT phase capillary column, Perkin-Elmer). Only end-point concentrations of N_2_O were above detection level for the root samples.

### DNA extraction and quantification of *nosZ* clades I and II gene abundances

DNA was extracted from soil and root samples using the FastDNA™ SPIN Kit for Soil (MP Biomedicals, Santa Ana, USA) following the manufacturer’s instructions with 200 or 300 mg of soil and roots, respectively. Whole root pieces were used for extraction of DNA from the root-associated compartment. In addition, DNA was extracted from 200 to 300 mg each of sterilized barley and sunflower seeds. The DNA concentration was determined using a Qubit^®^ fluorometer and the Qubit^®^ dsDNA BR assay kit (Life Technologies Corporation, Carlsbad, CA, USA).

To determine the genetic potential for N_2_O reduction, genes coding for the two known clades of N2O reductase, *nosZ* clade I and *nosZ* clade II, were quantified by quantitative real-time PCR (qPCR) using the primer pairs 1840F/2090R (Henry et al. 2006) and nosZ-II-F/nosZ-II-R (Jones et al. 2013). The qPCR reactions were performed in duplicate runs in a total reaction volume of 15 μl using 2X iQ™ SYBR Green supermix (BioRad, Hercules, CA, USA), 0.1% bovine serum albumin (New England Biolabs, Ipswich, MA, USA), primers (0.8 μM of each primer), and 10 ng DNA from the clayey soil (Ekhaga) and associated roots, and 2.5 ng DNA from the sandy soil (Kungshamn) and associated roots. The amplifications were done using the BioRad CFX Connect Real-Time System according to Hallin et al. (2015). Standard curves for each assay were generated by serial dilutions of linearized plasmids with cloned fragments of *nosZ*I from *Bradyrhizobium japonicum* USDA 110, and *nosZ*II from *Gemmatimonas aurantiaca* 27-T. Standard curves were linear (*R*^2^ = 0.99) in the range used. Amplification efficiencies were 81% and 76% for *nosZ*I and *nosZ*II, respectively. The amplifications were verified by melting curve analyses and agarose gel electrophoreses, and nontemplate controls resulted in negligible amplification. Potential inhibition in all samples was checked by amplifying a known amount of the pGEM-T plasmid (Promega) with the plasmid specific T7 and SP6 primers when added to the DNA extracts or nontemplate controls. PCR inhibitors were initially detected in DNA from Kungshamn soil and roots, but after additional dilution no inhibition of the amplification reactions was detected with the amount of DNA used.

### Sequencing of *nosZ* clades I and II amplicons and sequence processing

Sequences of *nosZ* clade I and *nosZ* clade II were PCR amplified from the DNA extracts in a two-step procedure. For the first PCR, three replicate reactions were performed using 10 ng template DNA, 1X Dream Taq Green PCR master Mix (Thermo Fisher Scientific, USA), 2.5 mM MgCl_2_, 0.1% BSA, and 0.8 μM of the *nosZI* primers CGCTSTTYMTIGAYAGYCAG and SKSACCTTITTRCCITYICG (Jones et al. 2014) or *nosZ* clade II primers (Jones et al. 2013) with 22 and 20 cycles of amplification for *nosZ* clades I and II, respectively. In the second PCR, 2 μl of the pooled PCR products from the first reaction were amplified in five replicates of a 15-cycle PCR using primers with sequencing key and adapter, with the forward primer including the barcodes. The final PCR products were agarose gel verified, pooled and purified using the QIAquick PCR Purification Kit (Qiagen, Marseille, France). Pyrosequencing was performed by Microsynth (Balgach, Switzerland) on a 454 GS FLX+ Genome Sequencer (Roche Applied Science, Penzberg, Germany).

Sequencing reads were screened and demultiplexed using QIIME (Caporaso et al. 2010) with default parameters. Subsequent processing was performed using a reference alignment and phylogeny of 441 full-length *nosZ* amino acid sequences as described in Graf et al. (2014). To remove nonspecific reads and reduce the occurrence of frame-shift errors, the HMM-FRAME algorithm (Zhang and Sun 2011) was used together with a hidden Markov model (HMM) based on the reference amino acid alignment. Chimeric sequences were removed by performing an initial screening using *de novo* detection of chimeras, followed by reference-based detection using the *nosZ* clade I and *nosZ* clade II databases obtained from FunGene (Fish et al. 2013). Sequences were then clustered into OTUs at 97% similarity with the ‘pick_otus.py’ method within QIIME, using the ‘usearch’ *de novo* OTU picking method. Representative sequences for each OTU were translated and aligned to the reference amino acid alignment using HMMER (Eddy 1998), and inspected using the ARB software (Ludwig et al. 2004). The final dataset consisted of 577 893 sequences (570–666 bp) after removal of singletons, resulting in an average of 4223 and 5856 sequences per sample clustered into 1243 and 2518 OTUs for clades I and II, respectively. Sequences are available at NCBI under BioProject accession number PRJNA314293.

### *nosZ* community diversity and composition

Species richness and Pielou’s evenness indices (Pielou 1966) were calculated for *nosZ* clades I and II communities in each sample. To account for differences in the number of sequences across replicates, 100 rarefactions of *nosZ*I and *nosZ*II OTU tables were generated using the ‘vegan’ package in the R environment (Oksanen et al. 2020) at sampling depths of 2677 and 3679 sequences, respectively, corresponding to the minimum number of sequences per sample in each table. The mean and standard deviation of each index was then calculated across all rarefactions for each replicate.

For phylogeny-based analysis, the FastTree algorithm (Price et al. 2010) was used to generate a maximum likelihood phylogeny (WAG substitution model; Whelan and Goldman 2001) based on the amino acid alignment of representative OTU sequences. Differences in the community structure among treatments were assessed by nonmetric multidimensional scaling of generalized UniFrac distance matrices (Chen et al. 2012) generated from *nosZ*I and *nosZ*II phylogenies and rarefied OTU tables, using the R packages ‘vegan’ and ‘GUniFrac.’ To identify OTUs that contributed significantly to changes in community structure between treatments, we used the Linear Decomposition Model (LDM) implemented in the R package ‘ldm,’ which tests for treatment effects over the whole community as well as amongst individual OTUs while controlling the false discovery rate (Hu and Satten 2020). Significance was determined by an omnibus test implemented in the ‘ldm’ packge that integrates three permutation tests of based on (i) relative abundances of OTUs, (ii) arcsine-root-transformed abundances, and (iii) presence–absence in rarefied OTU tables. This method accounts for variation in library size across samples, while providing greater consistency and sensitivity (Zhu et al. 2022). Analyses were performed separately for each clade and soil type, and the mean relative abundances of OTUs that were highly significant (*q* < 0.01) drivers of community structure were plotted as heatmaps using ‘ggtree’ (Yu 2020). Phylogenetic placement of reads and taxonomic assignment of OTUs was performed using the massively parallel evolutionary placement algorithm (EPA-NG) and gappa software (Barbera et al. 2019, Czech and Stamatakis 2019), with the *nosZ* database and phylogeny described above as a reference.

### Phylogenetic metrics for inferring community assembly processes

The net relatedness index (NRI) was calculated to examine the average phylogenetic distance between members within the communities, i.e. the relatedness among community members (Webb 2000). This metric can be used to infer the mechanisms for community assembly, since the outcome of various processes contributing to community assembly can be reflected in patterns of relatedness within the community assuming that the niches are nonrandomly distributed on the underlying phylogeny. Significant positive NRI-values indicate communities in which members are more phylogenetically related (clustered) than we could expect by chance, and here the environment has selected for organisms that share traits needed for that environment. By contrast, negative NRI-values describe communities that are more overdispersed across the phylogeny, and indicates competition as being important underlying process. The significance of phylogenetic clustering and overdispersion was determined using the trial-swap algorithm by Miklos and Podani (2004), with 999 permutations of communities randomly drawn from the entire metacommunity, which includes samples from all treatments in soil and root. A two-tailed test was used to determine the significance of NRI values at *P* = .05, in which observed ranks > 975 or < 25 indicated significant clustering or overdispersion, respectively. All calculations were performed using ‘picante’ (Kembel et al. 2010) and ‘EcoSimR’ (Gotelli and Ellison 2013) packages in R.

### Statistical analysis

All statistical analyses were performed using the R environment. Analysis of variance (ANOVA) and pairwise comparisons of treatments using Tukey’s honestly significant difference (HSD) test were performed in R. When underlying assumptions for ANOVA were violated, nonparametric rank-based tests were performed. Test of plant and soil effects on community composition based on the generalized UniFrac analyses were performed using a pairwise multiresponse permutation procedure (MRPP) with 999 permutations. Significance was corrected for multiple comparisons using the false discovery rate. To also assess the variability in genotype composition among treatments, the average distance from an individual unit to the group centroid in Euclidean space (beta-dispersion; Anderson et al. 2006) was calculated from GUnifrac ordinations using the R package ‘vegan.’ Two-way ANOVAs based on linear models were used to determine the main and interaction effects of plant species and soil type on diversity and gene abundances, and the proportion of variance explained (ω^2^) by each factor was calculated. Significance of treatment effect on community structure and proportion of explained variance by each factor were based on permutational multivariate ANOVA (perMANOVA) performed with the *adonis* function in ‘vegan’ using generalized Unifrac distances with *n* = 999 permutations.

## Results

### N_2_O reducing community composition

**Figure 1.**
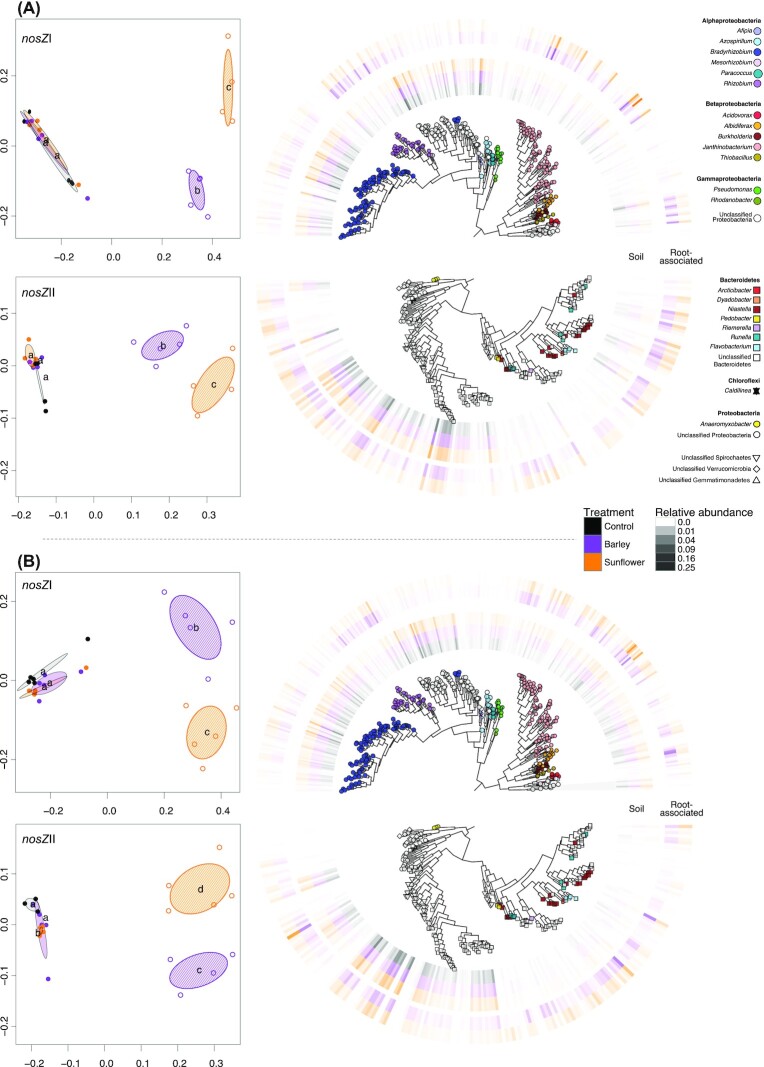
Soil and root-associated N_2_O reducing communities. Community structure of soil and root-associated *nosZ*I and *nosZ*II N_2_O reducing communities in (A) clay (Ekhaga) and (B) sandy (Kungshamn) soil types. Ordinations at left are based on nonmetric multidimensional scaling of generalized UniFrac distance metrics, with ellipses showing 95% confidence intervals within treatment groups and letters indicating significant differences between groups (FDR corrected-*P* < .05) based on a pairwise multiresponse permutation procedure. Open and closed symbols denote root-associated and soil communities, respectively, with orange = sunflower, purple = barley, and grey = unplanted soil. Phylogenies at right show *nosZ*I (upper) and *nosZ*II (lower) OTUs that significantly contributed to differences in community structure across treatments in each ordination, based on LDMs (FDR corrected *P* < .01). Outer rings show mean relative abundance of OTUs in each treatment and symbol shape and colour indicate taxonomic classification based on phylogenetic placement of OTUs within the reference *nosZ* phylogeny. Branch lengths of phylogenies have been square-root transformed for clarity.

The structure of both *nosZ* communities, based on generalized UniFrac distances, differed by soil type and plant species (Fig. 1; Figure S1, Supporting Information). Community structure was also significantly different between plant types in the root-associated communities. Both root-associated and soil communities in the sandy, acidic Kungshamn soils were distinct from those in the more clayey and neutral pH Ekhaga soil, which was confirmed by perMANOVA as soil type explained the largest proportion of variance in community structure estimated using generalized Unifrac distances (Table 1). In soil, the structure of both *nosZ* communities was determined only by soil type, explaining about 50% of the total variance in community structure. However, a significant plant effect (19.2%) was observed on the structure of the *nosZ*I root-associated communities. The variation in the structure of soil communities was lower than that of root-associated communities, which were more dispersed in the ordinations. This was confirmed by analysis of beta dispersion, which was significantly higher and more variable amongst root-associated *nosZ* communities compared to those in soil (Wilcoxon test, *P*< .001; Figure S2, Supporting Information). Phylogenetic placement of sequence reads of each *nosZ* variant within the *nosZ* reference phylogeny showed that in both soils, the majority of *nosZ*I organisms were associated with proteobacterial N_2_O reducers, whereas *nosZ*II communities consisted of a range of different taxa, primarily Bacteroidetes, Gemmatimonadetes, and Verrucomicrobia (Figures S3 and S4, Supporting Information).

**Table 1:** Proportion of variance explained (ω^2^) of the effect of soil type and plant species and their interaction on gene abundance ratios and diversity and community structure of *nosZ* clades I and II communities in root and soil samples.

		Root				Soil			
		Soil	Plant	Soil × plant	Residual	Soil	Plant	Soil × plant	Residual
**Abundance**	*nosZII/nosZI*	89.2***	-	3.2**	7.4	95.8***	1.0*	0.3	3.2
**Community**									
***nosZ*I**	Richness^1^	40.6**	-	1.2	58.2	84.7***	-	1.5	13.8
	Evenness^1^	52.0***	-	-	48.0	62.9***	-	-	37.1
	GUnifrac^2^	25.9***	19.2**	2.7	52.2	46.0***	-	-	54.0
***nosZ*II**	Richness^1^	-	-	-	100.0	68.2***	-	4.7	31.8
	Evenness^1^	49.9***	-	1.3	48.8	85.5***	-	3.9**	18.4
	GUnifrac^2^	45.2***	4.2	2.8	47.8	54.7***	0.6	1.6	43.1

1 Significance based on two-way ANOVA: * .01 < *P*< .05; ** .001 < *P*< .01; *** *P*< .001.

2 Significance of treatment effect and proportion of explained variance of generalized Unifrac distances based on perMANOVA.

Linear decomposition modelling identified 347 *nosZ*I and 204 *nosZ*II OTUs that were significant drivers of community separation between treatments in both soils (Fig. 1). Regardless of soil type, the separation between soil and root-associated *nosZ*I communities was mainly driven by a higher proportion OTUs identified as Betaproteobacterial and *Rhizobium nosZ*I in root-associated samples, whereas bulk soils had a higher relative abundance of *nosZ*I associated with other Alphaproteobacterial species, particularly *Bradyrhizobium* and *Azospirillum* (Fig. 1). In both soils, the separation between barley and sunflower root-associated communities was mainly driven by different lineages of Betaproteobacterial N_2_O reducing species, as the relative abundance of OTUs classified as *nosZ*I from *Acidovorax* was higher in barley root samples whereas several *Janthinobacterium nosZ*I OTUs were more abundant in sunflower roots. However, the relative abundance of OTUs classified as *Pseudomonas* and *Rhizobium nosZ*I was higher in barley roots in the sandy soil only (Fig. 1B). For the *nosZ*II communities, the separation between root-associated and soil communities was mainly driven by different lineages of Bacteroidetes, Spirochaetes, and Verrucomicrobia *nosZ*II OTUs, which were higher in abundance in root-associated communities for both soils, although this pattern was more pronounced in the clayey soil (Fig. 1B). By contrast, relative abundances of Chloroflexi and Gemmatimonadetes *nosZ*II OTUs were higher in soil communities for both soil types. Differences between sunflower and barley root-associated *nosZ*II communities were mainly driven by OTUs associated with various species of Bacteroidetes, which were typically found in greater relative abundance in barley root communities in both soils.

### Diversity and relatedness of N_2_O reducing communities

The *nosZ*II communities were more diverse than *nosZ*I across all samples (Table S1, Supporting Information). Root-associated *nosZ*I communities had lower richness and evenness compared to communities in the corresponding soil. This was not the case for clade II, as both richness and evenness were higher in the root-associated communities compared to those in soil compartment for the clayey soil, though only differences in evenness were significant. In the soil, richness of both clades was primarily affected by soil type with no influence of plants (Table 1). On the roots, richness of the *nosZ* clade I communities was determined by soil type with no significant effect of plant species, whereas *nosZ* clade II richness was unaffected by plant or soil type. However, the evenness of *nosZ* clade II soil communities was influenced by plant type depending on soil type, as indicated by the significant interaction term.

**Figure 2.**
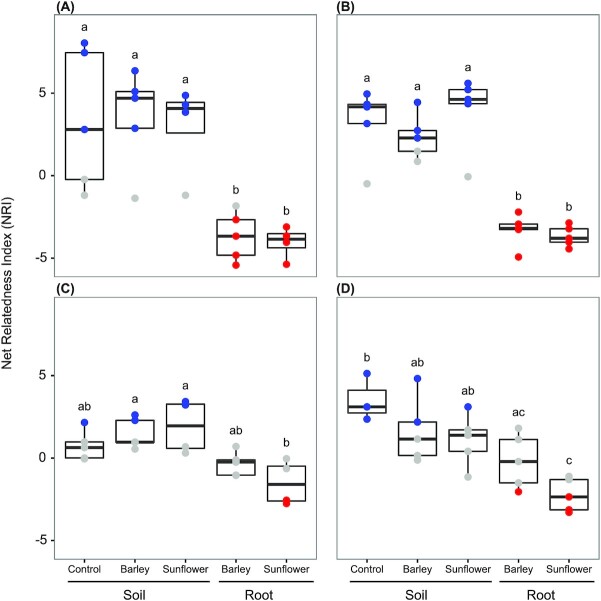
Phylogenetic relatedness of N_2_O reducing communities in soil and in association with roots of barley and sunflower, as well as in unplanted soil based on the NRI. (A) and (B) from clay (Ekhaga) and sandy (Kungshamn) soil types, and (C) and (D) *nosZ* clade II communities in clay and sandy soil types. The colour of the data points indicates if the communities are significantly phylogenetically clustered (blue) or overdispersed (red), or neutral (grey) based on null model simulations (*n* = 999). Letters above the boxes indicate significant differences between treatments (*P* < .05; *n* = 5).

Calculation of net-relatedness indices indicated that both clades I and II N_2_O root-associated communities were generally overdispersed, meaning that OTUs from these samples were more dispersed across the phylogeny than expected by chance, than those in the soil which were more clustered (Fig. 2). This trend was more pronounced in *nosZ* clade I communities, which exhibited significant overdispersion in roots of both plant species (Fig. 2A and B), whereas only a single clade II community from barley roots in the sandy soil was found to be significantly overdispersed (Fig. 2D).

### Gene abundances

Neither of the *nosZ* genes could be amplified using DNA extracted from the seeds despite that there was no PCR inhibition. This suggests that no microorganisms harbouring *nosZ* genes were present at detectable levels in the seed embryos. However, we cannot exclude that rare, undetected species could have influenced community composition during the experiment.

**Figure 3.**
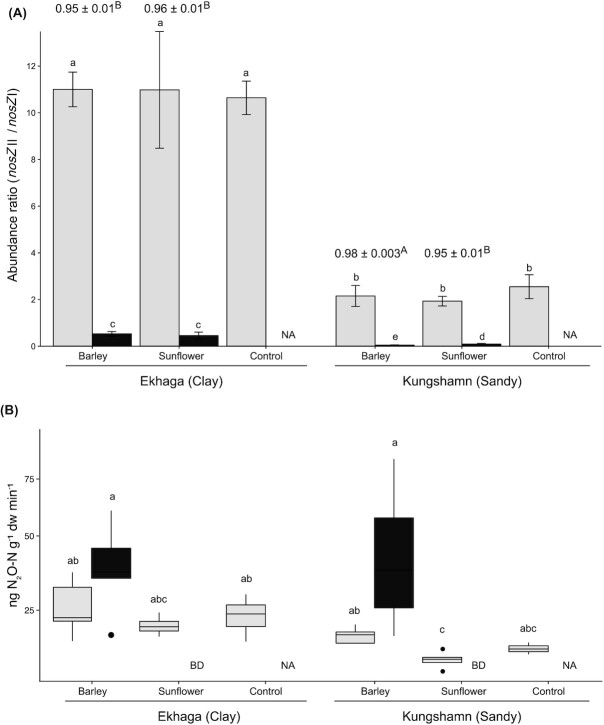
Abundances of *nosZ* genes and N_2_O production rates. (A) Abundance ratios of the genes *nosZ* clade II and *nosZ* clade I and (B) potential N_2_O production rates in soil (light grey boxes) in planted and unplanted (control) pots and in association with plant roots of barley and sunflower (dark grey boxes) from clayey (Ekhaga) and sandy (Kungshamn) soil types (mean ± SD; *n* = 5). Letters above the boxes indicate significant differences between treatments (*P*< .05). Numbers above treatments with plants in panel a show a normalized ratio of the gene abundances as the difference between abundance in the soil and root normalized to the soil ratio: ((*nosZ*II/*nosZ*I)_soil_-(*nosZ*II/*nosZ*I)_root_)/(*nosZ*II/*nosZ*I)_soil_.

There were significant differences in absolute abundances of *nosZ* clades I and II communities between samples (Table S2, Supporting Information). However, since abundances per g dry weight (DW) soil and root are not directly comparable, the ratios between *nosZ*II and *nosZ*I were used for comparison across all samples (Fig. 3A). The ratios were significantly higher in soil when compared to those in roots, showing that *nosZ* clade II and clade I communities dominate in different niches (*P*≤ .05). The ratio in both soil and root compartments was significantly affected by soil type, but plant species also played a role in root communities (Table 1). To compare the experimental treatments with plants on equal footing, we then calculated a normalized ratio of the gene abundances: ((*nosZ*II/*nosZ*I)_soil_-(*nosZ*II/*nosZ*I)_root_)/(*nosZ*II/*nosZ*I)_soil_, i.e. the difference between the soil and root normalized to the soil ratio (the maximal value) shown above the bars in Fig. 3(A). The higher the value for the normalized ratio, the stronger is the niche differentiation observed between the two clades. The normalized ratio was largely similar across soils and plant species and indicate only a slightly stronger separation in sandy soil with barely.

### Potential N_2_O production rates

Potential N_2_O production rates were similar across all soils, with the exception of sandy soil with sunflower that showed lower rates (Fig. 3B). In the root compartment of sunflower, no activity could be detected when growing in either of the two soils. By contrast, N_2_O production rates were higher in the root compartment of barley compared to soil in both soil types.

## Discussion

Both *nosZ* clades were present in the soil and root-associated communities. However, the *nosZ* gene abundance ratios indicate that populations of organisms carrying *nosZ* clade I were enriched in the root compartment for both plants, whereas clade II communities dominated in soil regardless of soil type and plant species. This, in conjunction with the finding that a comparatively large proportion of the *nosZ* clade I but not the clade II community structure could be explained by a plant factor, suggests that the two different N_2_O reducing communities occupy different ecological niches in the root–soil interface. The higher N_2_O production rates in barely roots compared to soil indicate higher availability of N_2_O. This could explain the relatively higher abundance of *nosZ* clade I in roots and *nosZ* clade II in soil as they have been suggested to have a lower apparent affinity for N_2_O (Yoon et al. 2016), although this is not conclusive (Conthe et al. 2018). However, this pattern was not observed for sunflower due to low or even inhibited activity of N_2_O producing microorganisms on sunflower roots. The difference in activity and abundance between barley and sunflower could be due to the inherent differences in root architecture and plant metabolites that give rise to distinct microbial community compositions for each plant. Roots in fibrous roots systems are thinner than those in tap root systems and root diameter controls the abundances of denitrifying bacteria and denitrification activities, with higher abundance and activity with decreasing root diameter (Legay et al. 2014, Cantarel et al. 2015, Moreau et al. 2015). Previous studies report allelopathic potential of sunflower roots and exudates (Ciarka et al. 2004), whereas barley exhibits substantial root exudate levels during the first 3–4 weeks of growth (Suku et al. 2014, Giles et al. 2017). This could also explain the high abundance of N_2_O reducers and N_2_O production activity in barley compared to sunflower. In agreement with our results, data showing selection of bacterial populations carrying *nosZ* clade I over clade II by roots from a range of plants is accumulating (Truu et al. 2017, Zhao et al. 2017, Ai et al. 2020), suggesting this could be a general feature. This is consistent with the root-associated environment being a ‘hotspot’ for denitrification and denitrifying microorganisms (Hamonts et al. 2013, Hallin et al. 2015, Graf et al. 2016, Moreau et al. 2019), as *nosZ* clade I is predominantly present in bacterial genomes with a complete denitrification pathway (Graf et al. 2014). Thus, based on our findings and those reported for other plant species, we propose that assembly of N_2_O reducers in the root-associated environment starts with selection for *nosZ* clade I bacteria over clade II. However, further work is required to determine if this is due to inherent characteristics of N_2_O reduction for energy conservation by organisms with clade I *nosZ*, or if it is due to other traits that are correlated with having either *nosZ* type.

Each soil constituted a unique pool of N_2_O reducing genotypes from which a selection was recruited to the root environment of barley and sunflower. In both soils, we observed an enrichment of *nosZ*I OTUs classified as *Acidovorax* and *Rhizobium* in barley root-associated communities. Species within these genera can produce compounds that promote the growth of barley in soil, although the abundance of *Rhizobium* species associated with barley roots has been shown to be dependent on plant genotype (Bziuk et al. 2021). Furthermore, the enrichment of *nosZ*II OTUs classified as Bacteroidetes in root-associated samples corresponds with previous culture- and 16S rRNA gene-based surveys on root-associated and rhizosphere communities (Johansen et al. 2009, Bulgarelli et al. 2015, Ling et al. 2022), and may be explained by the generally copiotrophic nature attributed to organisms within this phylum. The increased abundance in bulk soils of *nosZ*I OTUs classified as *Bradyrhizobium*, a genus shown to be common in endophytic bacterial communities of multiple barley cultivars (Yang et al. 2017), is also the most dominant bacterial lineage in soils throughout the world (Delgado-Baquerizo et al. 2018). They have large genomes encoding a vast diversity of functions—including denitrification—that allow them to persist as free-living diazotrophs in oligotrophic conditions (Poole et al. 2018, Tao et al. 2021). Oligotrophic conditions in bulk soil may also explain the greater abundance of *nosZ*II OTUs classified as Chloroflexi and Gemmatimonadetes, which in a recent meta-study have been shown to be depleted in the rhizosphere compared to bulk soil (Ling et al. 2022).

The difference in structure of the root-associated *nosZ* clade I and II N_2_O reducers and those in the soils was mainly driven by edaphic factors for both compartments. The higher abundance of *nosZ*II communities in the clayey, more neutral pH soil is in line with previous studies showing a general increase in *nosZ*II abundance with increasing pH (Jones et al. 2014, Samad et al. 2016). Differences in organic C and N also likely contribute to the uniqueness of N_2_O reducing communities in each soil type, with potentially higher availability of these resources in the clayey soil. The overriding effect of soil type is in line with what is known for microbial community structure in general in the root–soil continuum for different plant species (Berg and Smalla 2009, Bulgarelli et al. 2012, Lundberg et al. 2012, Edwards et al. 2015, Prasse et al. 2015) and recently reported also for *nosZ*I and *nosZ*II communities under cultivation of lucerne and cocksfoot (Graf et al. 2019). The root-associated N_2_O reducing communities were more variable in structure than those of the soil communities within each sample type, which could be an effect of subsampling the entire root system that comprises multiple niches and microbiomes (e.g. Liljerot et al. 1991, Baudoin et al. 2002). However, it could also indicate that stochastic recruitment processes played a role in the initial assembly process of root-associated *nosZ* communities, although this cannot be evidenced within this study. The root-associated N_2_O reducing communities, especially *nosZ* clade I, were less diverse than soil communities, yet more phylogenetically overdispersed, irrespective of soil type. Phylogenetic overdispersion has been shown to be indicative of competitive interactions having a stronger effect on community assembly, as opposed to more clustered phylogenetic patterns which imply selection of closely related organisms sharing advantageous traits for that particular habitat (Webb et al. 2002, Kraft et al. 2007). This suggest that closely related *nosZ* organisms are outcompeted by the remaining lineages, which may have traits that provide the greatest fitness within that niche, and that selection by environmental filtering plays a minor role in assembly of *nosZ*I N_2_O reducing communities in the root environment compared to *nosZ*II. The increased abundance of *nosZ* carrying taxa in root-associated samples that are commonly considered as copiotrophic, such as Bacteroidetes and Betaproteobacteria (Leff et al. 2015, Morrisey et al. 2016), lends further support to competition being an important driver of community assembly, as theses lineages may outcompete slower-growing or more specialized N_2_O reducing organisms when N_2_O is available as an electron acceptor. However, the presence of many niches or minimal niche overlap can also result in increased phylogenetic dispersion, which is a possible scenario in samples of whole plant roots.

Several studies suggest that extant microbial community assemblies are a result of a combination of both stochastic and deterministic processes (Burke et al. 2011, Caruso et al. 2011, Shafquat et al. 2014) where both temporal (Ferrenberg et al. 2013, Dini-Andreote et al. 2015) and spatial (Powell et al. 2015) scales need to be considered. Our observation is a snap-shot of the inferred assembly processes during the initial 3 weeks of plant growth in barley and sunflower roots, and we *a priori* established our scale of interest as being between the root system and the surrounding soil since we were interested in differences in assembly processes of N_2_O reducing communities at the overall root–soil interface. While the relative importance of different assembly mechanisms may differ at finer spatial or temporal resolution, we propose that an initial selection of *nosZ* clade I over clade II occurs in the root compartment simultaneously with a stochastic recruitment of genotypes within each clade, which would be followed by competition between the genotypes when the N_2_O reducers are establishing. A similar pattern was observed in a salt marsh chronosequence, in which the bacterial community composition was governed by stochasticity in the early successional stage and a progressive increase in deterministic selection was observed towards the late stages (Dini-Andreote et al. 2015). Niche-based theory also better explained assembly of rhizosphere communities after 5 years of soybean cultivation as compared with after 1 year (Mendes et al. 2014). Tkacz et al. (2015) showed that stochastic variation between replicates decreased when bacterial and fungal communities were monitored over three plant generations in microcosms, and by the third generation, replicate microcosms for each plant had communities that were more similar to each other for most plant species. For annual crops like sunflower and barley roots with a vegetation period of about 90–150 days, further research considering temporal effects on N_2_O-reducing root community assembly during the growth season is required to address if the initial processes are followed by other assembly processes and if initial effects remain.

The potential habitat separation between clades I and II and the different community assembly processes occurring in the root–soil interface can have functional consequences. Work on denitrifying microorganisms in pure cultures has suggested that species with a complete denitrification pathway may be more competitive in environments with low levels of nitrate, having the capacity to utilize all electron acceptors available from the reduction of nitrate (Felgate et al. 2012). This may explain the predominance of *nosZ* clade I organisms on both barley and sunflower roots in the present study given the competition from the plant for available nitrate (Moreau et al. 2015). From the same experiment as the one used in this study, we previously showed that the end-product rate ratio during denitrification (i.e. rate of N_2_O production in relation to total denitrification rate) in barley roots was 2–14 times lower in the root compartment compared to the soil (Graf et al. 2016), which confirms that denitrification is more efficient in close vicinity of roots. On the other hand, the higher product ratio in the soil would suggest that N_2_O production and reduction capacity are more loosely associated within the microbial community or that the denitrifier community simply maximizes energy conservation under the prevailing conditions and thereby terminate with N_2_O (van Spanning et al. 2007). This would indicate a higher risk for N_2_O emissions from soil communities compared to those associated with plant roots. Whether this can be generalized to other crops needs to be confirmed. In bulk soil, increased diversity and abundance of *nosZ* clade II, especially from Bacteroidetes, have been shown to play an important role for the soil N_2_O sink capacity (Jones et al. 2014). The majority of Bacteroidetes with *nosZ*II are likely to be nondenitrifying N_2_O reducers (Graf et al. 2014), which can reduce exogenous N_2_O produced by other organisms (Domeignoz-Horta et al. 2016). In the present study, different subclades in *nosZ* clade II related to *nosZ* in Bacteroidetes accounted for the separation between soil and root samples, which shows niche differentiation among N_2_O reducers within this phylum. However, the importance of their presence and *nosZ* clade II in general as N_2_O sinks at the root–soil interface remains elusive.

Overall, our study shows that niche-based selection and competition govern initial community assembly of N_2_O-reducing denitrifiers on roots of the annual crops barley and sunflower. The assembly processes were similar between the two crops and only marginally affected by soil type or plant species, although actual composition of the *nosZ* clades I and II communities depended on both these factors. The preferences of *nosZ* clades I and II bearing microorganisms for root and soil compartments, respectively, supports that those with *nosZ*II are subject to different environmental cues compared to N_2_O reducing denitrifiers (clade I), which should be investigated further and for other crops in order to possibly develop N_2_O-mitigation strategies in cropping systems.

## Supplementary Material

fiac092_Supplemental_file

## References

[bib1] Ai C , Zhang M, Sun Y et al. Wheat rhizodeposition stimulates soil nitrous oxide emission and denitrifiers harboring the nosZ clade I gene. Soil Biol Biochem. 2020;143:107738.

[bib2] Anderson MJ , Ellingsen KE, McArdle BH. Multivariate dispersion as a measure of beta diversity. Ecol Lett. 2006;9:683–93. 16706913 10.1111/j.1461-0248.2006.00926.x

[bib3] Barbera P , Kozlov AM, Czech L et al. EPA-ng: massively parallel evolutionary placement of genetic sequences. Syst Biol. 2019;68:365–9. 30165689 10.1093/sysbio/syy054 PMC6368480

[bib4] Baudoin E , Benizri E, Guckert A. Impact of growth stage on the bacterial community structure along maize roots, as determined by metabolic and genetic fingerprinting. Appl Soil Ecol. 2002;19:135–45.

[bib5] Berg G , Smalla K. Plant species and soil type cooperatively shape the structure and function of microbial communities in the rhizosphere. FEMS Microbiol Ecol. 2009;68:1–13. 19243436 10.1111/j.1574-6941.2009.00654.x

[bib6] Bowsher AW , Ali R, Harding SA et al. Analysis of wild sunflower (*Helianthus**annuus* L.) root exudates using gas chromatography-mass spectrometry. J Plant Nutr Soil Sci. 2015;178:776–86.

[bib83_1660658422001] Bulgarelli D , Garrido-Oter R, Munch PC et al. Structure and function of the bacterial root microbiota in wild and domesticated barley. Cell Host Microbe. 2015;17:392–403. 25732064 10.1016/j.chom.2015.01.011 PMC4362959

[bib7] Bulgarelli D , Rott M, Schlaeppi K et al. Revealing structure and assembly cues for *Arabidopsis* root-inhabiting bacterial microbiota. Nature. 2012;488:91–95. 22859207 10.1038/nature11336

[bib8] Burke C , Steinberg P, Rusch D et al. Bacterial community assembly based on functional genes rather than species. Proc Natl Acad Sci. 2011;108:14288–93. 21825123 10.1073/pnas.1101591108 PMC3161577

[bib82_955_165222] Bziuk N, Maccario L, Straube B et al. The treasure inside barley seeds: microbial diversity and plant beneficial bacteria . Environ Microbiome. 2021;16:20. 34711269 10.1186/s40793-021-00389-8 PMC8554914

[bib9] Cantarel AAM , Pommier T, Desclos-Theveniau M et al. Using plant traits to explain plant-microbe relationships involved in nitrogen acquisition. Ecology. 2015;96:788–99. 26236874 10.1890/13-2107.1

[bib10] Caporaso JG , Kuczynski J, Stombaugh J et al. QIIME allows analysis of high-throughput community sequencing data. Nat Methods. 2010;7:335–6. 20383131 10.1038/nmeth.f.303 PMC3156573

[bib11] Caruso T , Chan Y, Lacap DC et al. Stochastic and deterministic processes interact in the assembly of desert microbial communities on a global scale. ISME J. 2011;5:1406–13. 21368908 10.1038/ismej.2011.21 PMC3160679

[bib12] Chen J , Bittinger K, Charlson ES et al. Associating microbiome composition with environmental covariates using generalized unifrac distances. Bioinformatics. 2012;28:2106–13. 22711789 10.1093/bioinformatics/bts342 PMC3413390

[bib13] Ciarka D , Gawronska H, Malecka M et al. Allelopahtic potential of sunflower roots and root exudates. Zeszyty Problemowe Postepow Nauk Rolniczych. 2004;496:301–13.

[bib14] Conrad R. Soil microorganisms as controllers of atmospheric trace gases (H_2_, CO, CH_4_, OCS, N_2_O, and NO). Microbiol Rev. 1996;60:609–40. 8987358 10.1128/mr.60.4.609-640.1996 PMC239458

[bib15] Conthe M , Wittorf L, Kuenen G. et al. Growth yield and selection of *nosZ* clade II types in a continuous enrichment culture of N_2_O respiring bacteria. Envrion Microbiol Rep. 2018;10:239–44. 10.1111/1758-2229.12630 29457693

[bib16] Czech L , Stamatakis A. Scalable methods for analyzing and visualizing phylogenetic placement of metagenomic samples. PLoS ONE. 2019;14:e0219925. 31136592 10.1371/journal.pone.0217050 PMC6538146

[bib17] Delgado-Baquerizo M , Oliverio AM, Brewer TE et al. A global atlas of the dominant bacteria found in soil. Science. 2018;359:320–5. 29348236 10.1126/science.aap9516

[bib18] Dini-Andreote F , Stegen JC, van Elsas JD et al. Disentangling mechanisms that mediate the balance between stochastic and deterministic processes in microbial succession. Proc Natl Acad Sci. 2015;112:E1326–32. 25733885 10.1073/pnas.1414261112 PMC4371938

[bib20] Domeignoz-Horta LA , Putz M, Spor A et al. Non-denitrifying nitrous oxide-reducing bacteria - an effective N2O sink in soil. Soil Biol Biochem. 2016;103:376–9.

[bib19] Domeignoz-Horta LA , Spor A, Bru D et al. The diversity of the N_2_O reducers matters for the N_2_O:N_2_ denitrification end-product ratio across an annual and a perennial cropping system. Front Microbiol. 2015;6:698. 26441904 10.3389/fmicb.2015.00971 PMC4585238

[bib21] Eddy SR Profile hidden Markov models. Bioinformatics. 1998;14:755–63. 9918945 10.1093/bioinformatics/14.9.755

[bib22] Edwards J , Johnson C, Santos-Medellín C et al. Structure, variation, and assembly of the root-associated microbiomes of rice. Proc Natl Acad Sci USA. 2015;112:201414592. 10.1073/pnas.1414592112 PMC4345613 25605935

[bib23] Felgate H , Giannopoulos G, Sullivan MJ et al. The impact of copper, nitrate and carbon status on the emission of nitrous oxide by two species of bacteria with biochemically distinct denitrification pathways. Environ Microbiol. 2012;14:1788–800. 22642644 10.1111/j.1462-2920.2012.02789.x

[bib24] Ferrenberg S , O'Neill SP, Knelman JE et al. Changes in assembly processes in soil bacterial communities following a wildfire disturbance. ISME J. 2013;7:1102–11. 23407312 10.1038/ismej.2013.11 PMC3660671

[bib25] Fish JA , Chai B, Wang Q et al. FunGene: the functional gene pipeline and repository. Front Microbiol. 2013;4:1–14. 24101916 10.3389/fmicb.2013.00291 PMC3787254

[bib27] Giles CD , Brown LK, Adu MO et al. Response-based selection of barley cultivars and legume species for complementarity: root morphology and exudation in relation to nutrient source. Plant Sci. 2017;255:12–28. 28131338 10.1016/j.plantsci.2016.11.002

[bib31] Gotelli NJ , Ellison AM. EcoSimR 1.00. 2013. http://www.uvm.edu/∼ngotelli/EcoSim/EcoSim.html. Last accessed November 2016.

[bib28] Graf DRH , Jones CM, Hallin S. Intergenomic comparisons highlight modularity of the denitrification pathway and underpin the importance of community structure for N_2_O emissions. PLoS ONE. 2014;9:1–20. 10.1371/journal.pone.0114118 PMC4250227 25436772

[bib30] Graf DRH , Saghaï A, Zhao M et al. Lucerne (*Medicago**sativa*) alters N_2_O-reducing communities associated with cocksfoot (*Dactylis**glomerata*) roots and promotes N_2_O production in intercropping in a greenhouse experiment. Soil Biol Biochem. 2019;137:107547.

[bib29] Graf DRH , Zhao M, Jones CM et al. Soil type overrides plant effect on genetic and enzymatic N_2_O production potential in arable soils. Soil Biol Biochem. 2016;100:125–8.

[bib32] Hallin S , Hellman M, Choudhury MI et al. Relative importance of plant uptake and plant associated denitrification for removal of nitrogen from mine drainage in sub-Arctic wetlands. Water Res. 2015;85:377–83. 26360231 10.1016/j.watres.2015.08.060

[bib33] Hallin S , Philippot L, Löffler FE et al. Genomics and ecology of novel N_2_O-reducing microorganisms. Trends Microbiol. 2018;26:43–55. 28803698 10.1016/j.tim.2017.07.003

[bib34] Hamonts K , Clough TJ, Stewart A et al. Effect of nitrogen and waterlogging on denitrifier gene abundance, community structure and activity in the rhizosphere of wheat. FEMS Microbiol Ecol. 2013;83:568–84. 23006139 10.1111/1574-6941.12015

[bib35] Henry S , Bru D, Stres B et al. Quantitative detection of the *nosZ* gene, encoding nitrous oxide reductase, and comparison of the abundances of 16S rRNA, *narG*, *nirK*, and *nosZ* genes in soils. Appl Environ Microbiol. 2006;72:5181–9. 16885263 10.1128/AEM.00231-06 PMC1538733

[bib36] Henry S , Texier S, Hallet S et al. Disentangling the rhizosphere effect on nitrate reducers and denitrifiers: insight into the role of root exudates. Environ Microbiol. 2008;10:3082–92. 18393993 10.1111/j.1462-2920.2008.01599.x

[bib37] Hu YJ , Satten GA. Testing hypotheses about the microbiome using the linear decomposition model (LDM). Bioinformatics. 2020;36:4106–15. 32315393 10.1093/bioinformatics/btaa260 PMC8453243

[bib38] Johansen JE , Nielsen P, Binnerup SJ. Identification and potential enzyme capacity of flavobacteria isolated from the rhizosphere of barley (*Hordeum vulgare* l.). Can J Microbiol. 2009;55:234–41. 19370066 10.1139/w08-116

[bib39] Jones CM , Graf DRH, Bru D et al. The unaccounted yet abundant nitrous oxide-reducing microbial community: a potential nitrous oxide sink. ISME J. 2013;7:417–26. 23151640 10.1038/ismej.2012.125 PMC3554408

[bib40] Jones CM , Spor A, Brennan FP et al. Recently identified microbial guild mediates soil N_2_O sink capacity. Nat Clim Change. 2014;4:1–5.

[bib41] Kembel SW , Cowan PD, Helmus MR et al. Picante: R tools for integrating phylogenies and ecology. Bioinformatics. 2010;26:1463–4. 20395285 10.1093/bioinformatics/btq166

[bib42] Kraft NJB , Cornwell WK, Webb CO et al. Trait evolution, community assembly, and the phylogenetic structure of ecological communities. Am Nat. 2007;170:271–83. 17874377 10.1086/519400

[bib81_1660657185656] Kuzyakov Y , Blagodatskaya E. Microbial hotspots and hot moments in soil: Concept & review. Soil Biol Biochem. 2015;83:184–199.

[bib43] Langarica-Fuentes A , Manrubia M, Giles ME et al. Effect of model root exudate on denitrifier community dynamics and activity at different water-filled pore space levels in a fertilised soil. Soil Biol Biochem. 2018;120:70–9.

[bib84_1660658982590] Leff JW , Jones SE, Prober SM et al. Consistent responses of soil microbial communities to elevated nutrient inputs in grasslands across the globe.Proc Natl Acad Sci. 2015;112:10967–10972. 26283343 10.1073/pnas.1508382112 PMC4568213

[bib44] Legay N , Baxendale C, Grigulis K et al. Contribution of above- and below-ground plant traits to the structure and function of grassland soil microbial communities. Ann Bot. 2014;114:1011–21. 25122656 10.1093/aob/mcu169 PMC4171078

[bib45] Liljeroth E , Burgers SLGE, Van Veen JA. Changes in bacterial populations along roots of what (*Triticum**aestivum* L.) seedlings. Biol Fertil Soils. 1991;10:276–80.

[bib46] Ling N , Wang T, Kuzyakov Y. Rhizosphere bacteriome structure and functions. Nat Commun. 2022;13:836. 35149704 10.1038/s41467-022-28448-9 PMC8837802

[bib47] Ludwig W , Strunk O, Westram R et al. ARB: a software environment for sequence data. Nucleic Acids Res. 2004;32:1363–71. 14985472 10.1093/nar/gkh293 PMC390282

[bib48] Lundberg DS , Lebeis SL, Paredes SH et al. Defining the core *Arabidopsis**thaliana* root microbiome. Nature. 2012;488:86–90. 22859206 10.1038/nature11237 PMC4074413

[bib49] Mendes LW , Kuramae EE, Navarrete AA et al. Taxonomical and functional microbial community selection in soybean rhizosphere. ISME J. 2014;8:1577–87. 24553468 10.1038/ismej.2014.17 PMC4817605

[bib50] Miklos I , Podani J. Randomization of presence–absence matrices: comments and new algorithms. Ecology. 2004;85:86–92.

[bib52] Moreau D , Bardgett RD, Finlay RD et al. A plant perspective on nitrogen cycling in the rhizosphere. Func Ecol. 2019;33:540–52.

[bib51] Moreau D , Pivato B, Bru D et al. Plant traits related to nitrogen uptake influence plant-microbe competition. Ecology. 2015;96:2300–10. 26405754 10.1890/14-1761.1

[bib85_1660659278876] Morrissey EM , Mau RL, Schwartz E et al. Phylogenetic organization of bacterial activity. ISME J. 2016;10:2336–2340. 26943624 10.1038/ismej.2016.28 PMC4989319

[bib53] Oksanen J , Blanchet G, Friendly M et al. Vegan: Community Ecology Package. 2020. https://CRAN.R-project.org/package=vegan Last accessed March 2021.

[bib54] Philippot L , Andert J, Jones CM et al. Importance of denitrifiers lacking the genes encoding the nitrous oxide reductase for N_2_O emissions from soil. Glob Change Biol. 2011;17:1497–504.

[bib55] Philippot L , Hallin S, Börjesson G et al. Biochemical cycling in the rhizosphere having an impact on global change. Plant Soil. 2009;321:61–81.

[bib56] Philippot L , Hallin S, Schloter M. Ecology of denitrifying prokaryotes in agricultural soil. Adv Agron. 2007;96:249–305.

[bib58] Pielou EC. The measurement of diversity in different types of biological collections. J Theor Biol. 1966;13:131–144.

[bib60] Poole P , Ramachandran V, Terpolilli J. Rhizobia: from saprophytes to endosymbionts. Nat Rev Microbiol. 2018;16:291–303. 29379215 10.1038/nrmicro.2017.171

[bib59] Powell JR , Karunaratne S, Campbell CD et al. Deterministic processes vary during community assembly for ecologically dissimilar taxa. Nat Commun. 2015;6:8444. 26436640 10.1038/ncomms9444 PMC4600744

[bib61] Prasse CE , Baldwin AH, Yarwood SA. Site history and edaphic features override the influence of plant species on microbial communities in restored tidal freshwater wetlands. Appl Environ Microbiol. 2015;81:3482–91. 25769832 10.1128/AEM.00038-15 PMC4407224

[bib62] Price MN , Dehal PS, Arkin AP. FastTree 2 - approximately maximum-likelihood trees for large alignments. PLoS ONE. 2010;5:e9490. 20224823 10.1371/journal.pone.0009490 PMC2835736

[bib64] Samad MS , Biswas A, Bakken LR et al. Phylogenetic and functional potential links pH and N_2_O emissions in pasture soils. Sci Rep. 2016;6:35990 27782174 10.1038/srep35990 PMC5080606

[bib65] Sanford RA , Wagner DD, Wu Q et al. Unexpected nondenitrifier nitrous oxide reductase gene diversity and abundance in soils. Proc Natl Acad Sci. 2012;109:19709–14. 23150571 10.1073/pnas.1211238109 PMC3511753

[bib66] Shafquat A , Joice R, Simmons SL et al. Functional and phylogenetic assembly of microbial communities in the human microbiome. Trends Microbiol. 2014;22:261–6. 24618403 10.1016/j.tim.2014.01.011 PMC4008634

[bib67] Shcherbak I , Millar N, Robertson GP. Global meta-analysis of the nonlinear response of soil nitrous oxide (N_2_O) emissions to fertilizer nitrogen. Proc Natl Acad Sci. 2014;111:9199–204. 24927583 10.1073/pnas.1322434111 PMC4078848

[bib68] Suku S , Knipfer T, Fricke W. Do root hydraulic properties change during the early vegetative stage of plant development in barley (*Hordeum**vulgare*)?. Ann Bot. 2014;113:385–402. 24287810 10.1093/aob/mct270 PMC3906963

[bib69] Tao JJ , Wang SS, Liao TH et al. Evolutionary origin and ecological implication of a unique nif island in free-living bradyrhizobium lineages. ISME J. 2021;15:3195–206. 33990706 10.1038/s41396-021-01002-z PMC8528876

[bib70] Tkacz A , Cheema J, Chandra G et al. Stability and succession of the rhizosphere microbiota depends upon plant type and soil composition. ISME J. 2015;9:2349–59. 25909975 10.1038/ismej.2015.41 PMC4611498

[bib71] Truu M , Ostonen I, Preem J-K et al. Elevated air humidity changes soil bacterial community structure in the silver birch stand. Front Microbiol. 2017;8:557 28421053 10.3389/fmicb.2017.00557 PMC5376589

[bib72] van Spanning RJM , Richardson DJ, Ferguson SJ. Introduction to the biochemistry and molecular biology of denitrification. In: Bothe H, Ferguson SJ, Newton WE (eds). Biology of the Nitrogen Cycle. Amsterdam: Elsevier, 2007, 3–20.

[bib74] Webb CO , Ackerly DD, Mcpeek M a et al. Phylogenies and community ecology. Ann Rev Ecol Syst. 2002;33:475–505.

[bib73] Webb CO. Exploring the phylogenetic structure of ecological communities: an example for rain forest trees. Am Nat. 2000;156:145–55. 10856198 10.1086/303378

[bib75] Whelan S , Goldman N. A general empirical model of protein evolution derived from multiple protein families using a maximum-likelihood approach. Mol Biol Evol. 2001;18:691–9. 11319253 10.1093/oxfordjournals.molbev.a003851

[bib77] Yang J , Pan X. Root exudates from sunflower (*Helianthus**annuus* L.) show a strong adsorption ability toward Cd(II). J Plant Interact. 2013;8:263–70.

[bib78] Yang L , Danzberger J, Shöler A et al. Dominant groups of potentially active bacteria shared by barley seeds become less abundance in root associated microbiome. Front Plant Sci. 2017;8:1005. 28663753 10.3389/fpls.2017.01005 PMC5471333

[bib79] Yin C , Fan X, Yan G et al. Gross N_2_O production process, not consumption, determines the temperature sensitivity of net N_2_O emission in arable soil subject to different long-term fertilization practices. Front Microbiol. 2020;11:745 32411109 10.3389/fmicb.2020.00745 PMC7198778

[bib80] Yoon S , Nissen S, Park D et al. Nitrous oxide reduction kinetics distinguish bacteria harboring clade I NosZ from those harboring clade II nosZ. Appl Environ Microbiol. 2016;82:3793–800. 27084012 10.1128/AEM.00409-16 PMC4907195

[bib81] Yu G. Using ggtree to visualize data on tree-like structures. Curr Protic Bioinfoormatics. 2020;69:e96. DOI: 10.1002/cpbi.96. 10.1002/cpbi.96 32162851

[bib82] Zhang Y , Sun Y. HMM-FRAME: accurate protein domain classification for metagenomic sequences containing frameshift errors. BMC Bioinf. 2011;12:198. 10.1186/1471-2105-12-198 PMC3115854 21609463

[bib83] Zhao M , Jones CM, Meijer J et al. Intercropping affects genetic potential for inorganic nitrogen cycling by root-associated microorganisms in *Medicago**sativa* and *Dactylis**glomerata*. Appl Soil Ecol. 2017;119:260–6.

[bib84] Zhu ZY , Satten GA, Hu YJ. Integrative analysis of relative abundance data and presence-absence data of the microbiome using the LDM. Bioinformatics. 2022;38:1–3. 10.1093/bioinformatics/btac181 PMC9113255 35561163

