# Supplementary material for: Assembly of root-associated N2O-reducing communities of annual crops is governed by selection for nosZ clade I over clade II
Source: FEMS Microbiol Ecol. 2022 Aug 4;98(9):fiac092. doi: 10.1093/femsec/fiac092 (PMC9397574; doi:10.1093/femsec/fiac092)
Supplement: fiac092_Supplemental_file [file fiac092_supplemental_file.pdf]

Supplementary data

Assembly of root-associated N_2O-reducing communities of annual crops is governed by selection for *nosZ* clade I over clade II

Daniel R.H. Graf, Christopher M. Jones, Ming Zhao, Sara Hallin

Table S1. Diversity and absolute abundance of *nosZ* clade I and clade II in soil and root compartments.

Table S2. Absolute abundance of *nosZ* clade I and clade II in soil and root compartments.

Fig. S1 Ordinations of generalized UniFrac distance matrices using non-metric multidimensional scaling of *nosZ* Clade I and *nosZ* Clade II communities in soil and roots.

Fig. S2. Distribution of beta dispersion for N_2O reducing communities in soil and in association with roots of barley and sunflower, as well as in unplanted soil.

Fig. S3. Phylogenetic placement of total *nosZ* clade I and clade II sequences from soil and root associated communities in the clayey (Ekhaga)soil.

Fig. S4. Phylogenetic placement of total *nosZ* clade I and clade II sequences from soil and root associated communities in the sandy (Kungshamn) soil.

Table S1. Diversity and absolute abundance of *nosZ* clade I and clade II (copies per g^{-1} dry weight (DW) of soil) in soil and root compartments for each treatment (mean ± SD, n=5). Letters indicate significant differences between treatments (P < 0.05, Tukey's HSD test)

			**Nitrous oxide reducers**			
			***nosZ* clade I**		***nosZ* clade II**	
**Soil Type**	**Compartment**	**Plant**	**(Richness)[1]**	**(Evenness)[2]**	**(Richness)[1]**	**(Evenness)[2]**
Ekhaga	Soil	Barley	252^{b} ± 27	0.73^{b} ± 0.02	424^{b} ± 23	0.74^{c} ± 0.01
(Clay)		Sunflower	273^{ab} ± 24	0.73^{b} ± 0.02	402^{b} ± 26	0.71^{c} ± 0.01
		Unplanted control	280^{ab} ± 26	0.74^{b} ± 0.03	436^{b} ± 21	0.74^{c} ± 0.02
	Root	Barley	204^{c} ± 28	0.62^{c} ± 0.05	462^{ab} ± 26	0.84^{a} ± 0.01
		Sunflower	171^{c} ± 34	0.60^{c} ± 0.08	475^{ab} ± 18	0.85^{a} ± 0.01
Kungshamn	Soil	Barley	382^{a} ± 12	0.79^{a} ± 0.01	512^{a} ± 36	0.80^{b} ± 0.01
(Sandy)		Sunflower	366^{a} ± 12	0.79^{a} ± 0.01	518^{a} ± 24	0.80^{b} ± 0.00
		Unplanted control	371^{a} ± 9	0.79^{a} ± 0.01	483^{ab} ± 17	0.79^{bc} ± 0.00
	Root	Barley	243^{bc} ± 29	0.73^{b} ± 0.04	452^{ab} ± 32	0.80^{b} ± 0.02
		Sunflower	245^{bc} ± 24	0.74^{b} ± 0.05	467^{ab} ± 23	0.79^{bc} ± 0.04

[1]OTU richness
[2]Pielou's evenness

Table S2. Absolute abundance of *nosZ* clade I and clade II (copies per g^{-1} dry weight (dw) of soil) in soil and root compartments (copies per g^{-1} dw of root material) for each treatment (mean ± SD, n=5). letters indicate significant differences between treatments (p < 0.05, Tukey's HSD test) in either soil (lowercase) or root compartments (uppercase).

			***nosZ* clade I**	***nosZ* clade II**
**Compartment**	**Soil Type**	**Plant**	**($\times 10^8$ g^{-1} DW)**	**($\times 10^8$ g^{-1} DW**
Soil	Ekhaga	Barley	0.46^{a} ± 0.15	4.97^{a} ± 1.57
	(Clay)	Sunflower	0.43^{a} ± 0.10	4.72^{a} ± 1.23
		Unplanted control	0.47^{a} ± 0.09	4.91^{a} ± 0.79
	Kungshamn	Barley	0.63^{a} ± 0.07	1.34^{b} ± 0.24
	(Sandy)	Sunflower	0.56^{a} ± 0.07	1.07^{b} ± 0.14
		Unplanted control	0.58^{a} ± 0.25	1.41^{b} ± 0.50
Root	Ekhaga	Barley	3.15 ± 0.94^{C}	1.69^{A} ± 0.69
	(Clay)	Sunflower	4.69 ± 1.39^{C}	2.10^{A} ± 0.75
	Kungshamn	Barley	41.72 ± 18.01^{A}	1.89^{A} ± 0.96
	(Sandy)	Sunflower	13.98 ± 7.74^{B}	1.11^{A} ± 0.33

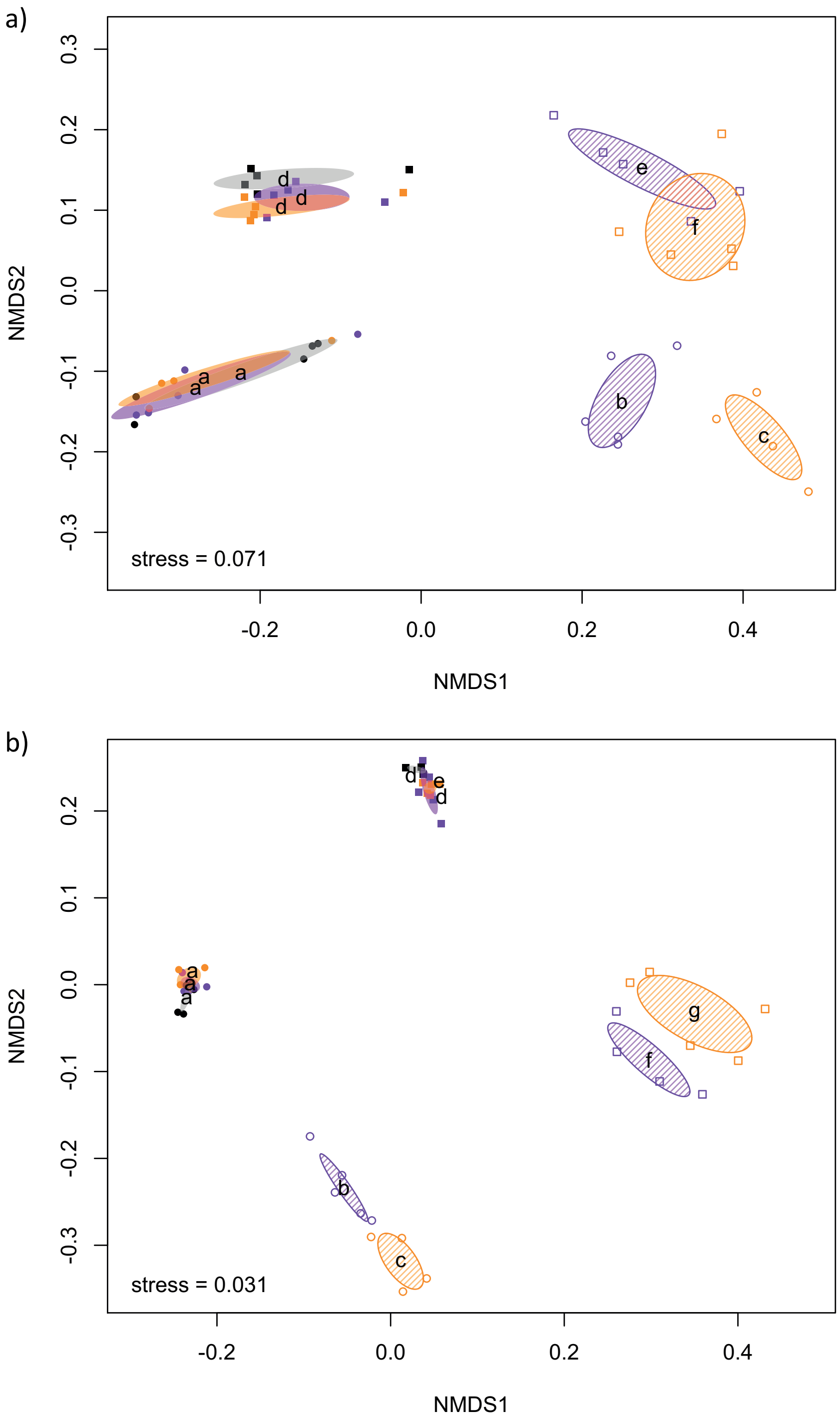

Fig. S Ordinations of generalized UniFrac distance matrices using non-metric multidimensional scaling of (**a**) *nosZ* Clade I and (**b**) *nosZ* Clade II communities in soil (closed symbols) and roots (open symbols). Symbols represent pots with clayey soil (Ekhaga, circles) and sandy soil (Kungshamn, squares). Colors indicate pots with barley (purple), sunflower (orange), and unplanted soil (black). Ellipses show 95 % confidence intervals. Letters denote significant differences between groups based on a pairwise multi-response permutation procedure, with significance corrected for multiple comparisons ($P < 0.05$).

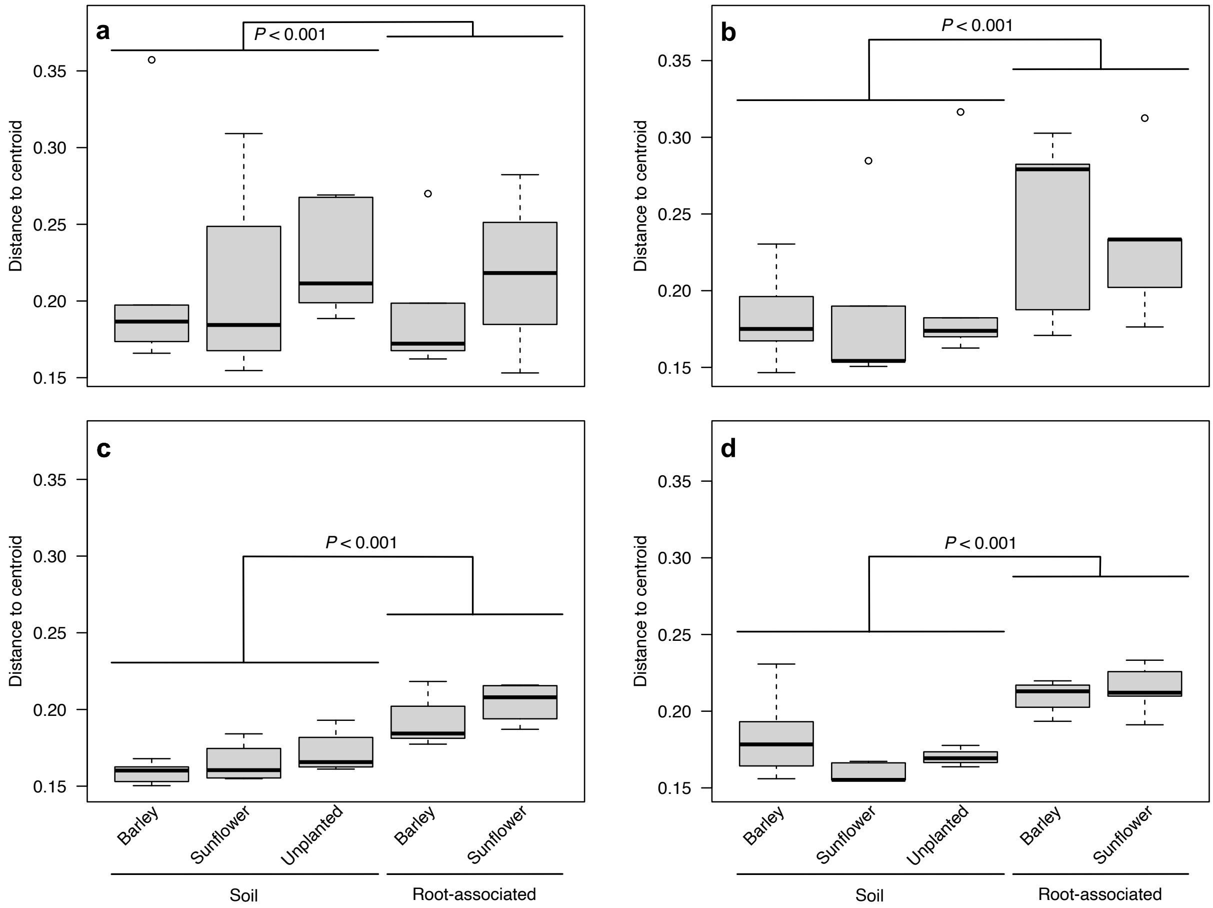

Fig. S2. Distribution of beta dispersion for N_2O reducing communities in soil and in association with roots of barley and sunflower, as well as in unplanted soil. (**a**) *nosZ* clade I communities in clay soil (Ekhaga) (**b**) *nosZ* clade I communities in sandy soil (Kungshamn), (**c**) *nosZ* clade II communities in Ekhaga and (**d**) *nosZ* clade II communities in Kungshamn shown as the distances between samples within each treatment to the centroid in Euclidean space. Circles indicate outlier data points. Significant differences in betadispersal between root and soil communities are indicated (Wilcoxon test).

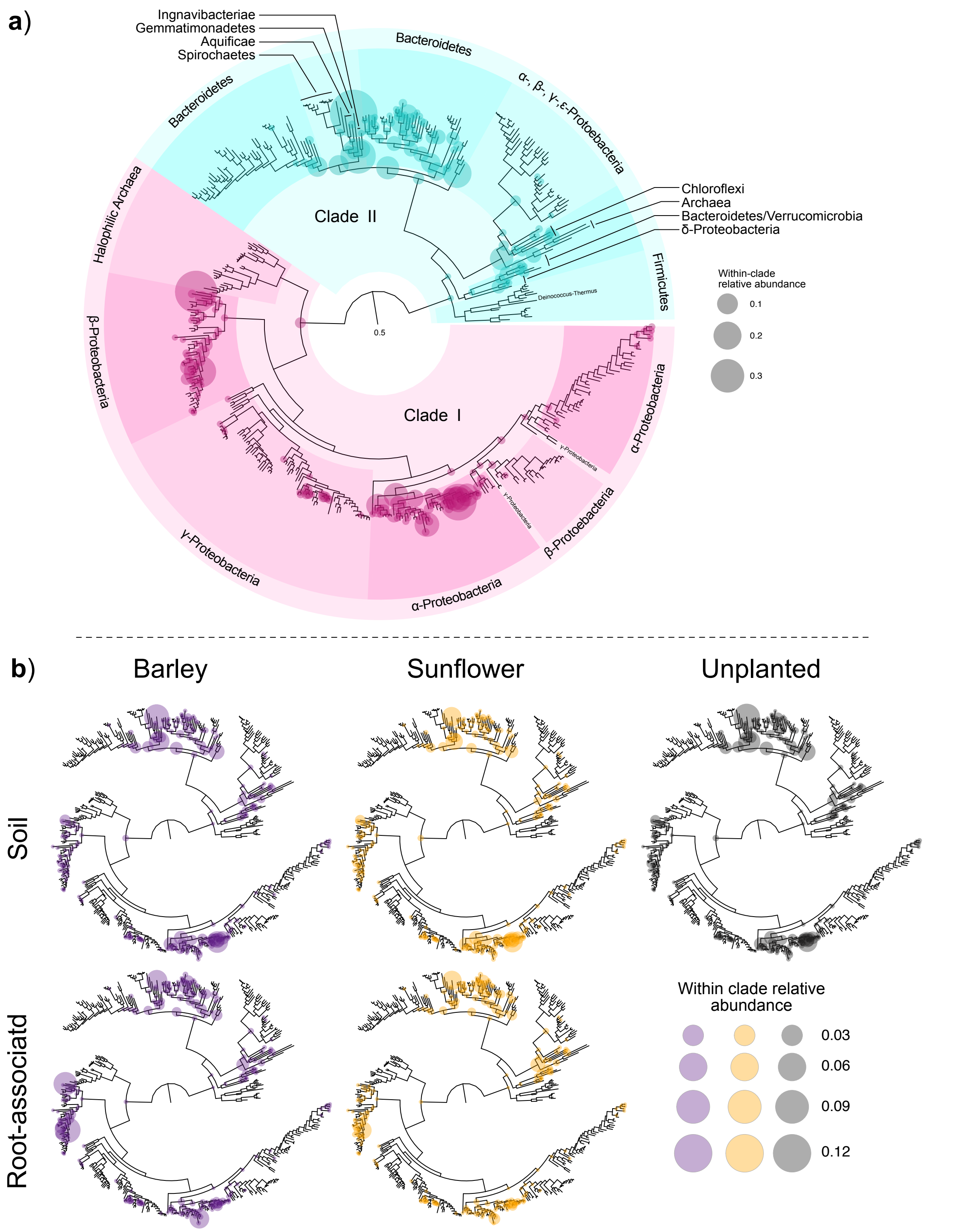

Fig. S3. (**a**) Phylogenetic placement of total *nosZ* clade I and clade II sequences from soil and root associated communities in the **clayey (Ekhaga)** soil within the reference phylogeny (maximum likelihood; LG+Γ substitution model) of 441 *nosZ* full-length amino acid sequences. Groupings of reference *nosZ* sequences within major taxonomic classifications are shaded, and branch length of the phylogeny is indicated by the scale bar at center. Circle size indicates the within-clade relative abundances of reads placed at each node. (**b**) Placement of reads within the reference phylogeny across soil and root associated communities for each plant type. Circle size indicates the within-clade relative abundances of reads placed at each node within each clade for each combination of community and treatment.

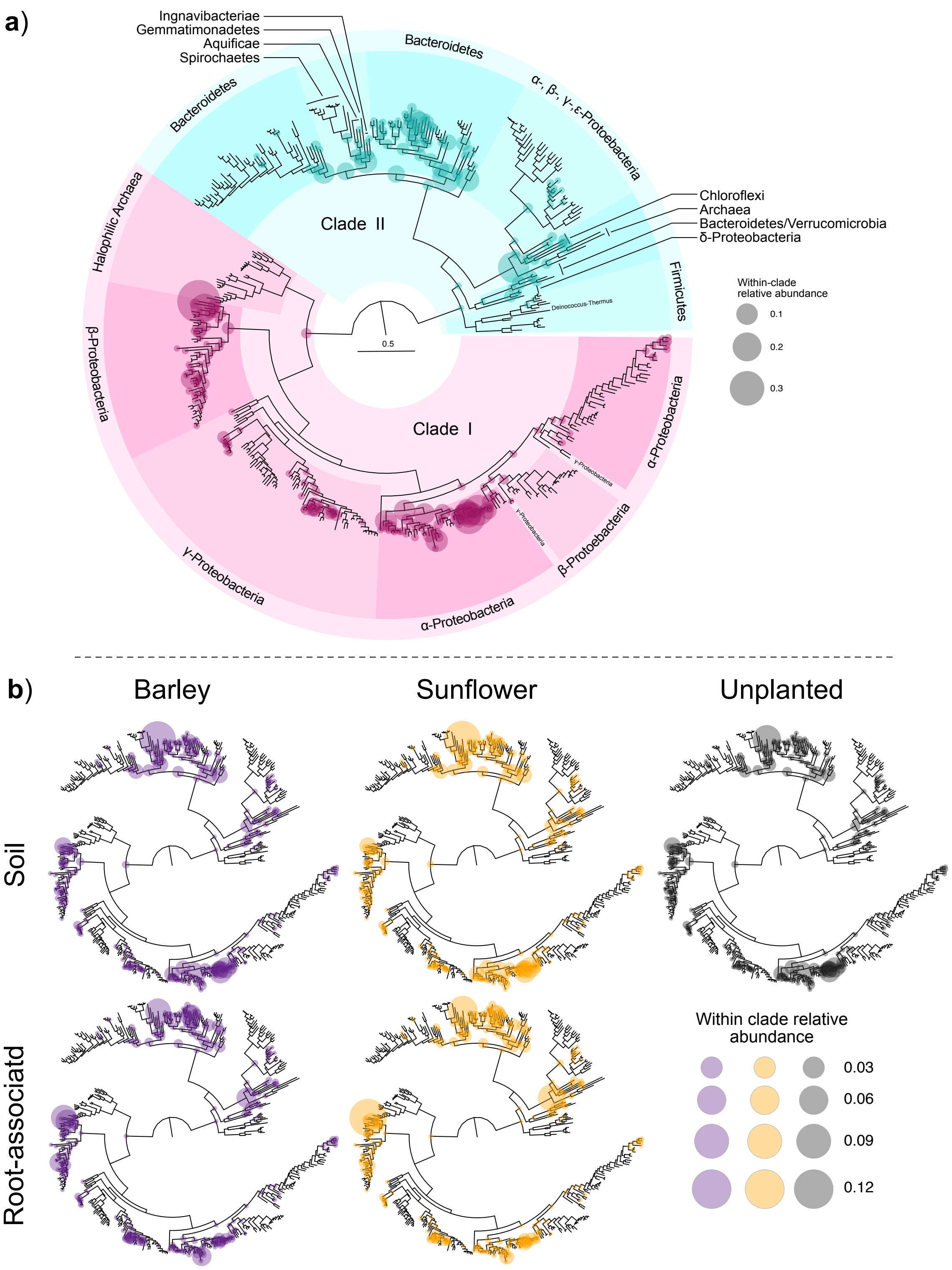

Fig. S4. (**a**) Phylogenetic placement of total *nosZ* clade I and clade II sequences from soil and root associated communities in the **sandy (Kungshamn)** soil within the reference phylogeny (maximum likelihood; LG+Γ substitution model) of 441 *nosZ* full-length amino acid sequences. Groupings of reference *nosZ* sequences within major taxonomic classifications are shaded, and branch length of the phylogeny is indicated by the scale bar at center. Circle size indicates the within-clade relative abundances of reads placed at each node. (**b**) Placement of reads within the reference phylogeny across soil and root associated communities for each plant type. Circle size indicates the within-clade relative abundances of reads placed at each node within each clade for each combination of community and treatment.
